# Renewal MI Dental Composite Etch and Seal Properties

**DOI:** 10.3390/ma15155438

**Published:** 2022-08-08

**Authors:** Nabih Alkhouri, Wendy Xia, Paul F. Ashley, Anne M. Young

**Affiliations:** 1 Department of Biomaterials and Tissue Engineering, UCL Eastman Dental Institute, London NW3 2QG, UK; 2 Department of Paediatric Dentistry, UCL Eastman Dental Institute, London WC1E 6DE, UK

**Keywords:** dental composite, microleakage, dentine sealing, enzymatic degradation, remineralization, minimally invasive dentistry

## Abstract

This study’s aim was to assess whether the Renewal MI composite can self-etch enamel, seal sound cavities, and stabilize demineralized dentine. Etching was assessed using scanning electron microscopy (SEM). Cavity sealing was quantified using the ISO-11405 dye microleakage test. Demineralized dentine stabilization was evaluated by visualizing resin tag formation, enzyme activity and mineral precipitation at the adhesion interface. Renewal MI provided a mild etching of sound enamel in comparison with 37% phosphoric acid. It provided a comparable seal of sound cavities to Z250/Scotchbond Universal adhesive and a superior seal to Activa, Fuji IX and Fuji II LC. With demineralized dentine, Renewal MI formed 300–400 µm resin tags covering 63% of the adhesion interface compared with 55 and 39% for Z250/Scotchbond and Activa. Fuji IX and Fuji II LC formed no resin tags. A higher tag percentage correlated with lower surface enzyme activity. Unlike Activa and Fuji II LC, Renewal MI promoted mineral precipitation from simulated body fluid, occluding adjacent dentinal tubules within 6 months. These novel etching and sealing properties may facilitate Renewal MI’s application in minimally invasive dentistry.

## 1. Introduction

Dental caries is caused by specific oral bacteria (e.g., *Streptococcus mutans* and *lactobacillus*) producing organic acids that demineralize the enamel and then the underlying dentine [1]. Enzyme-catalysed hydrolysis and solubilization of demineralized dentine by matrix metalloproteinases (MMPs) subsequently occurs [2]. Caries is one of the most common human diseases worldwide, with disadvantaged groups disproportionately affected [3]. In 2019, the average percentage of 5-year-old children with caries was 23% in England (up to 34% in more deprived areas). Only 10% of teeth with caries in dentine were restored [4,5]. Untreated caries can progress to pulpitis, periapical pathology, and the need for tooth extraction. In the UK, this is currently the main reason for a child’s hospital admission [4].

The difficulty of providing dental care to anxious or pre-cooperative children is one of the most common reasons for them not to have their teeth restored. Silver–mercury amalgam fillings were the mainstay of treatment for many years. They are strong, simple to place and antibacterial [6,7]. Following the global Minamata agreement (Minamata convention on mercury, UN, 2013), however, all mercury-containing products are being phased out globally. Consequently, the use of amalgam in children was banned in July 2018 across the EU [8].

The main alternative filling material is the dental composite. Unlike amalgam, composites are not antibacterial. Instead, they rely on an effective cavity seal, restricting bacterial growth. For conventional composites to work, multiple complicated steps must be followed to ensure effective adhesion to the tooth structure [9,10]. Prior to composite placement (as with amalgam), the total removal of dental caries is necessary [11]. This generally requires local anaesthesia and high-speed drilling. Acid-etching and rinsing are then required to provide a surface to which the composite adhesive can bond. Ideally, placement is carried out under rubber dam isolation to prevent saliva/bacterial recontamination. Unfortunately, all of these steps are particularly difficult for children to cope with. This can make placement either impossible or significantly compromised. Consequently, these fillings can suffer a high failure rate, largely due to recurrent disease (secondary caries) at the restoration/tooth interface [12,13,14,15].

With the advent of the global COVID-19 pandemic, composite placement became even more difficult due to the multiple drilling and washing steps being aerosol-generating procedures (AGPs), which can increase the risk of disease spread [16]. Modern concepts of caries management that support minimally invasive techniques may reduce this risk and the difficulties in treating children [17]. These techniques keep as much tooth tissue as possible and may reduce or eliminate the need for local anaesthetic injections and drilling. Following effective cavity sealing, the residual underlying bacteria decline in number, and the demineralized tooth structure re-hardens [18]. Poor composite restoration sealing, however, would allow nutrient and water ingress. These processes potentially enable residual bacterial growth, continuing enzyme action, bond damage and then further bacterial invasion.

Glass ionomer cements (GICs) are often cited as being more suitable for use in children’s teeth. This is due to the greater ease of placement and use with minimally invasive methods. GICs contain an aqueous polyacid solution that enables self-etching and bonding to the tooth structure. Unfortunately, their low strength can mean that the longevity and clinical performance with larger cavities are poor [19,20]. To overcome these issues, many different composite/GIC hybrid formulations, including compomers and resin modified glass ionomer cements (RMGICs), have been developed. These, however, still often require either tooth acid etching/conditioning and/or bonding steps to ensure effective retention [21].

Renewal MI is a new experimental flowable hybrid composite developed at the UCL Eastman Dental Institute and licensed to Schottlander UK. It was developed primarily for children but may benefit from other applications such as special care dentistry. Renewal MI has the potential to be used in a simplified one-step restorative procedure. It may be placed directly on affected dentine without extensive caries removal, etchant, or adhesive use. Renewal MI has an added adhesion-promoting monomer to improve bonding to the tooth structure. Additionally, calcium phosphate particles are included that react with surface water to produce acid for self-etching. The reaction also provides minerals to support long-term restoration self-repair through their precipitation at the adhesion interface. Furthermore, Renewal MI contains hydrophilic polylysine particles that may further absorb water from the tooth surface, enabling subsequent sealing by the otherwise hydrophobic resin phase. It is expected to also reduce the possibility of enzyme-activated dentine hydrolysis.

The aim of this paper was therefore to assess the etching of enamel by Renewal MI versus phosphoric acid. Renewal MI sealing (without etching and bonding), of sound enamel and dentine cavities was then determined. A commercial composite (placed with etch and adhesive), two RMGICs and a GIC were used as comparators. Furthermore, resin tag formation and enzyme deactivation at material/demineralized dentine interfaces were compared. Finally, mineral precipitation at the interface between Renewal MI or the RMGICs and demineralized dentine was assessed following 6 months of restoration immersion in simulated dentinal fluid (SBF).

The null hypotheses for quantitative tests were that there is no significant difference between Renewal MI and other commercial comparators in terms of:

1. sealing sound drilled cavities.
2. forming resin tags and inhibiting enzyme activity with demineralized dentine.

Renewal MI’s ability to etch enamel in comparison to phosphoric acid and precipitate minerals was assessed qualitatively.

## 2. Materials and Methods

### 2.1. Materials

#### 2.1.1. Restorative Materials

The main chemical components of the materials investigated in this study are summarised in Table 1. Renewal MI (Davis, Schottlander and Davis Dental Company, Letch-worth, UK) was supplied in compules. The conventional composite Filtek Z250 (3M ESPE, St. Paul, MN, USA) employed with Scotchbond Universal etchant gel (37% phosphoric acid, 3M ESPE, St. Paul, MN, USA), and Universal Scotchbond adhesive. The GIC used was Fuji IX GP (GC America, Alsip, IL, USA). The RMGICs investigated were Fuji II LC (GC America, Alsip, IL, USA) and Activa Kids (Pulpdent, Watertown, MA, USA).

#### 2.1.2. Teeth

Teeth were obtained from the Eastman Dental Institute biobank. The study has been approved by the UCL Eastman Biobank Ethics Committee under a generic project ethical approval number 1304 (first dated 29 April 2014). Primary molars were used in drilled cavity studies, as this was the population and tooth type of main interest. Permanent teeth, however, were used in etching and demineralized dentine investigations. This was because extracted primary teeth are usually deeply decayed. Conversely, sound adult premolars and molars may be extracted for orthodontic reasons. They therefore provided sufficiently large depth of sound dentine required for the demineralized dentine studies.

### 2.2. Enamel Etching

To compare the effect of Renewal MI versus phosphoric acid gel on enamel etching, flat enamel surfaces were obtained by grinding down the buccal surface of human permanent teeth using 500-grit abrasive paper. Teeth were then shaken in water for 5 min (Jintai^®^ JT-14 Dental Lab Round Shaker Oscillator, Jentai, Zhejiang, China) to ensure the removal of any debris. Enamel surfaces were blot dried and Renewal MI or phosphoric acid gel applied for 20, 40, 60, 90 or 120 s. Phosphoric acid was rinsed off with water. Renewal MI was dissolved using acetone and removed by vortexing for 1 min followed by vigorous washing with water for a further minute. The etched enamel surfaces were blow-dried, sputter-coated with gold/palladium and visualized through scanning electron microscopy (SEM, Phillip XL-30, Eindhoven, The Netherlands).

### 2.3. Drilled Cavity Restoration and Dye Microleakage

#### 2.3.1. Tooth Restoration

Microleakage at the drilled tooth–restoration interface was investigated following the instructions of ISO/TS 11405:2015 [22] using primary molars. As access to primary teeth was limited, to maximize sample numbers, multiple cylindrical cavities were drilled in each tooth. Cavities were 2 mm in diameter and 2 mm deep for enamel leakage assessment. For dentine leakage assessment (with the surface enamel removed), the depth was increased to 3 mm. Seventy teeth were used, with 3 or 4 cavities per tooth. Only non-carious buccal, lingual, or proximal surfaces at the middle third were used. Where possible, each tooth had one cavity restored with each of the different restorative materials. Cavities were restored with the different materials after the following tooth preparation steps:

- Renewal MI, Fuji II LC or Fuji IX: directly without cavity preconditioning [23].
- Activa: acid etching for 10 s, then rinse and dry (5 s using an air syringe).
- Z250: acid etching (30 s enamel, 15 s dentine), then rinse and dry. Bonding agent was applied and light-cured for 20 s following manufacturer’s instructions.

Renewal MI, Z250 and Fuji II LC were light-cured for 20 s (Demi Plus, Kerr, Orange, CA, USA, wavelength range: 450–470 nm, intensity from 1100–1330 mW/cm).

Restorations were carefully polished (using composite polishing bur) to ensure the removal of any excess material on the outer enamel surface. For the assessment of leakage at the dentine/restoration interface with no enamel, cavity depth was increased to 3 mm. The restoration was then polished down to remove the top ~1 mm-thick surface of both enamel and material and expose the dentine/material interface. Restored teeth were then incubated in water at 37 °C for 24 h prior to dye leakage assessment.

#### 2.3.2. Dye Microleakage Evaluation

Before immersion in dye, apices of the above teeth were sealed with adhesive wax. The rest of the tooth surface was covered with 2 layers of nail varnish (except for the top surface of the fillings and 1 mm around them). Specimens were subsequently placed in aqueous methylene blue (1%) for 4 h to allow dye penetration through any gaps at the adhesion interface. Following rinsing and drying, teeth were carefully cross sectioned longitudinally to expose each restoration/tooth interface, one at a time. These were examined under a light microscope and dye microleakage scored as follows:

- 0: None
- 1: Within enamel (only used for cavities with enamel cavosurface)
- 2: In dentine but did not reach the pulpal wall
- 3: Reached the pulpal wall

When the results were more variable (e.g., for the RMGICs), sample numbers were increased to enhance confidence in any differences with those for Renewal MI. Sample numbers were: for Renewal MI, 18 and 26, Z250, 11 and 11, Activa, 30 and 31, Fuji IX, 16 and 16 and for Fuji II LC, 27 and 37 for enamel and dentine, respectively. Data are reported as both the number with each score and this number as a percentage of the group number. For example, for Renewal MI, numbers are provided as a percentage of 18 or 26 for enamel versus dentine, respectively.

### 2.4. Demineralized Dentine Sealing, Enzyme Activity and Remineralization

#### 2.4.1. Dentine Disc Demineralization

Discs of demineralized dentine were prepared by first removing the occlusal enamel from sound permanent human teeth using a cutting machine (Leica SP1600, Leica Biosystems, Milton Keynes, UK) and then obtaining the top 2 mm-thick slice of coronal dentine. These discs were immersed in 15 mL of formic acid (4M) for 2 days to achieve total demineralization. Complete demineralization without collapse of the dentine tubule structure was demonstrated through SEM with EDX (Inca X-Sight 6650 detector, Oxford Instrument, Abingdon, UK). The final discs were flexible, with tubules from the top to the lower surfaces providing a porous mesh-like structure composed largely of collagen [24].

#### 2.4.2. Resin Tag Formation

For material/demineralized dentine penetration assessment (resin tag formation), pastes were confined within circlips of 10 mm internal diameter and 1 mm thickness. Circlips were placed on top of an acetate sheet. Pastes included Renewal MI, Z250, Activa, Fuji IX and Fuji II LC. The above demineralized dentine discs were then pressed on the top surfaces of the uncured material pastes. For Z250, Scotchbond was pre-applied to the dentine surface prior to dentine placement in contact with the composite. Materials requiring light exposure were then exposed to the above LED light for 40 s on each side. The light tip was placed in direct contact with acetate or demineralized dentine. To visualize the paste’s ability to penetrate the demineralized dentine, the collagen was completely dissolved by immersion in sodium hypochlorite (15%) for 2 days. This exposed any polymerized resin tags formed by material penetration into the demineralized dentine mesh.

Relatively high-magnification SEM images (×100–500) were used to enable visualization of localized tags density and length. Additionally, lower-magnification images (×40) of the whole collagen imprint area were used to quantify total surface coverage percentage. Lower-magnification images demonstrated that regions containing tags seen at a high magnification could be patchy. Imprint areas were significantly darker in regions of high resin tag density. ImageJ software was used to determine the percentage of the collagen surface that had been sealed by tags. This was achieved through first determining the total adhesion area (dentine/material contact area with the central pulp horn region subtracted). The remaining imprint dark and lighter regions were converted to black and white, respectively (*n* = 3 per material). Tag coverage was defined as the percentage of the adhesion area that was black.

Additionally, restored demineralized dentine discs (*n* = 3) were polished using 500-grit abrasive paper to expose the adhesion interface. The exposed interface was then stained with Rhodamine B (0.2% in isopropanol, Sigma, Welwyn Garden City, UK) for 2 min followed by a gentle rinse. This enabled the visualization of resin tags within tubules using confocal laser scanning microscopy (CLSM Bio-Rad Confocal Microscope/Olympus BX51 Upright Microscope, Olympus Corporation, Tokyo, Japan). An oil objective lens (×60) and the red fluorescence channel were used. The laser microscope settings for rhodamine B were 568 nm excitation and emission through 600–630 nm filter. The Z motor was used to acquire 35 scans of the same field up to 18 µm deep at 2 µm intervals. These scans were compiled using ImageJ software to enable visualising an 18 µm depth of the interface in a 2D image.

#### 2.4.3. Enzyme Activity

To assess enzyme activity at the demineralized dentine/restorative material interface, a Molecular Probe (EnzChek Collagenase Assay Kit, Thermo Fisher Scientific, Waltham, MA, USA) was employed. This kit contains a denatured collagen/fluorescein conjugate that, when degraded, produces strong green fluorescence. In this study, 5 µL of the kit collagen substrate was diluted 1:4 with buffer to obtain a collagen concentration of 200 µg/mL. The diluted collagen probe was applied to the blot dried collagen disc surfaces and left for 5 min to soak in. The probe-soaked surface was then restored, as in the resin tag test above, using Renewal MI and commercial restorative material pastes (*n* = 3). A negative control of non-restored demineralized dentine was used. Polymerization, when required for setting, was initiated through exposure of the lower surfaces to LED light for 40 s. Set restorations were stored in a humid sealed pot with a moist tissue in a 37 °C incubator. Samples were polished down to expose the interface after 1 day or 2 weeks to enable scanning through CLSM. Samples were orientated to scan a representative cross-section region of the adhesion interface. The selected field was scanned using a ×60 lens and focused to gain the highest fluorescence possible without detecting the weak collagen green auto fluorescence. Images were analysed using ImageJ software v1.8 (NIH, LOCI, University of Wisconsin, Madison, WI, USA). Relative MMP activity was taken as the area percentage of the scanned field which had strong green fluorescence.

#### 2.4.4. Mineral Precipitation at Set Material/Demineralized Dentine Interfaces

The above demineralized dentine discs were also used on set materials to determine their tooth remineralization potential. Renewal MI, Fuji II LC and Activa pastes were placed in 10 mm-internal diameter and 1 mm-thick circlips sandwiched between 2 acetate sheets. Set discs were obtained by the 40 s exposure of the top and bottom surfaces to the above LED light placed in direct contact with the acetate sheet. Following set disc removal from the mould, demineralized dentine discs, prepared as above, were gently clamped against the material surfaces (*n* = 3). Samples were then fully immersed in 10 mL of simulated body fluid (SBF) prepared according to ISO 23317:2014. Samples were incubated at 37 °C, with the SBF being replaced every 2 weeks. After 6 months, discs were gently detached, and the dentine interface imaged by SEM.

### 2.5. Statistical Analysis

All values and error bars reported were the mean with 95% confidence intervals (95% CI). SPSS Statistics v24 for Windows (IBM, Armonk, NY, USA) was used for statistical analysis. For dye leakage, ordinal regression and Kruskal–Wallis pairwise comparisons were undertaken. For tag coverage and enzyme activity area, Levene’s test was used to assess the homogeneity of variance. When variances were equal, data were analysed using one-way analysis of variance (ANOVA) followed by Tukey’s post hoc test for multiple comparisons when needed. The Kruskal–Wallis test was used if the variances were not equal, followed by pairwise comparisons if needed [25]. The significance value was *p* = 0.05.

## 3. Results

### 3.1. Enamel Etching

**Figure 1:**
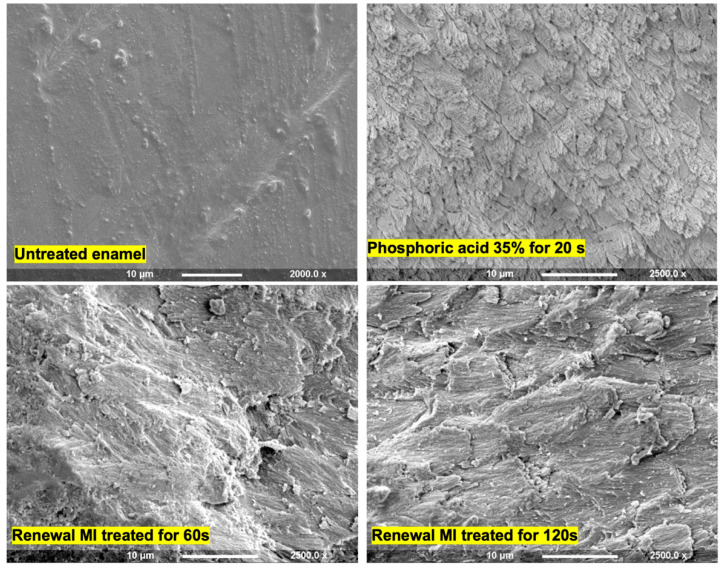
Representative SEM images of the enamel surface upon applying Renewal MI or phosphoric acid gel 37% for different times followed by rinsing with acetone and/or water.

Enamel exposed to Renewal MI showed a roughened uneven surface in comparison to untreated ground enamel (Figure 1). The etching pattern observed, however, was different from that seen with phosphoric acid etching. It also required longer exposure times (60 instead of 20 s) to become well-defined.

### 3.2. Dye Microleakage in Sound Cavities

**Figure 2:**
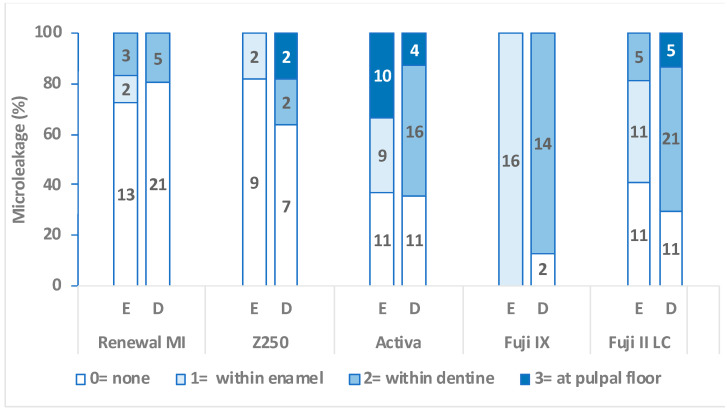
Blue dye microleakage following immersion in water for 24 h at 37 °C. The labels on the bars indicate the numbers of cavities. The *y* axis indicates this number as a percentage of the total group sample number. The cavosurface was either enamel (E) or dentine (D). The darker the column, the more extensive the dye penetration, and the worse the cavity sealing.

Blue dye microleakage results with enamel or dentine cavosurfaces are shown in Figure 2. The darker the column, the deeper the dye penetration. Statistical analysis showed that microleakage with enamel or dentine cavities restored with Z250 (with etch and bond) and Renewal MI were comparable to each other. Both were significantly lower compared with all other commercial comparators (*p* = 0.01 to 0.007).

### 3.3. Sealing of Demineralized Dentine

#### 3.3.1. Tags Formation

**Figure 3:**
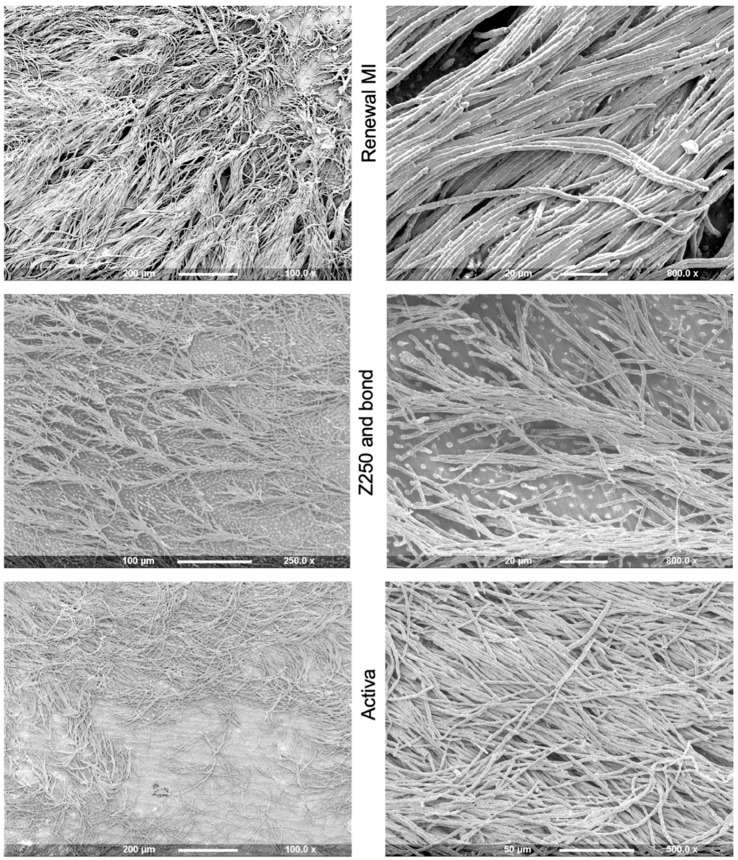
SEM images of resin tags formed within demineralized dentine by Renewal MI, Z250 and Activa (Fuji II LC and Fuji IX gave no tags).

**Figure 4:**
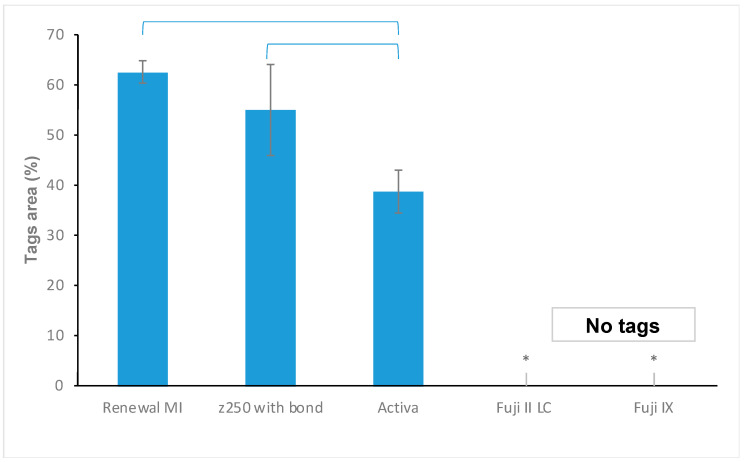
Tag coverage area at the adhesion interface with the demineralized dentine using Renewal MI, Z250, Activa, Fuji IX or Fuji II LC. * indicates a significant difference from all other groups, while the blue bars indicate a significant difference within groups. Error bars are 95% CI (*n* = 3).

Example SEM images of resin tags formed within demineralized dentine after collagen dissolution are provided in Figure 3. Some tag-free areas were detected often around the edge of the collagen imprint area or surrounding the central pulp horn areas. Percentages of the collagen imprint area covered by tags are provided in Figure 4.

Renewal MI showed an extensive network of 300–400 µm-long tags (Figure 3) covering 62% (Figure 4) of the demineralized dentine adhesion surface. Whilst imprint surface area covered by tags by the Z250 bonding agent (55%) was not significantly different, the tags within these areas were clearly shorter and less densely packed. In comparison, Activa tags were of intermediate length but covered significantly less (39%) of the surface (*p* = 0.034 to 0.001). Fuji II LC and Fuji IX provided no detectable resin tags. In the latter case, the use of sodium hypochlorite was questionable, as it caused Fuji IX to soften, as well as dissolve collagen.

**Figure 5:**
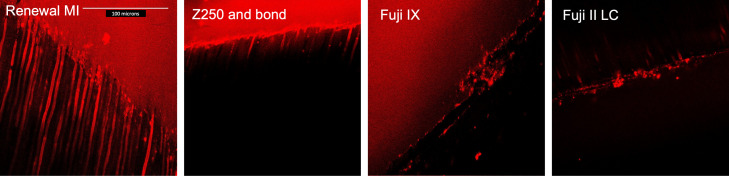
CLSM images of resin tags formation within demineralized dentine by Renewal MI, Z250, Fuji IX or Fuji II LC. All images are of areas 204 µm × 204 µm.

The CLSM scans (Figure 5), however, could help address this concern. As seen with SEM, tags formed using Renewal MI were much longer in comparison to those of the Z250 Scotchbond adhesive. With Z250, a few microns-thick adhesive layer was detectable beneath the resin tags. Fuji IX and Fuji II LC, however, showed minimal evidence for material tubule penetration and a less well-defined interface.

#### 3.3.2. Enzyme Activity in Restored Demineralized Dentine

**Figure 6:**
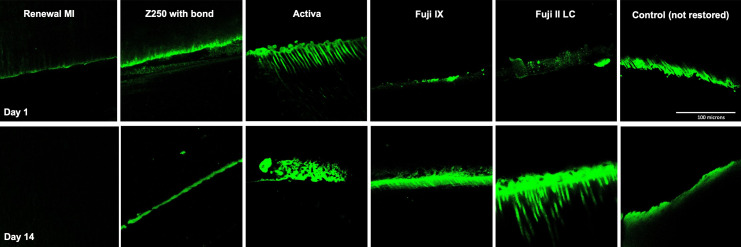
Representative CLSM images (204 × 204 µm) demonstrating MMP activity (in green) after 1 day and 14 days at the adhesion interface between Renewal MI, Z250 with bonding agent, Activa, Fuji IX or Fuji II LC and demineralized dentine. Non-restored demineralized dentine was used as a negative control.

Representative example CLSM images of green fluorescence due to the collagenase molecular probe at the interface between materials and demineralized dentine discs are provided in Figure 6. Renewal MI showed the least fluorescence at the adhesion interface, which disappeared almost totally after 14 days. For other materials, the fluorescence was mostly seen as a band at the interface, although in Fuji II LC and Activa it could additionally be observed penetrating down into the dentine tubules.

**Figure 7:**
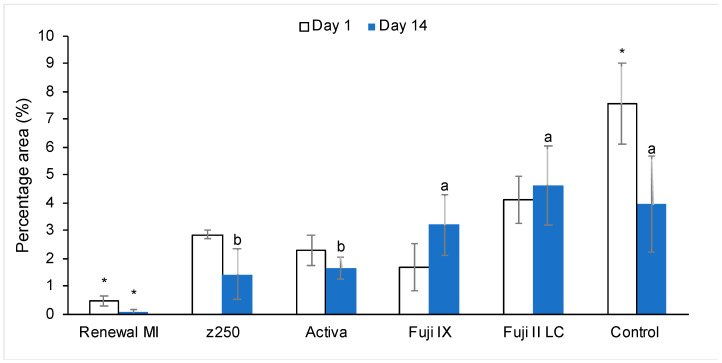
Green fluorescence area as a percentage of CLSM images of enzyme activity. This indicates the relative level of interface MMP activity on demineralized dentine restored with different materials (1 or 14 days). * indicates significant difference from all other groups. Sharing the same letter (a or b) means no significant difference between these groups. Error bars are 95% CI (*n* = 3).

Figure 7 provides green fluorescence area percentages obtained using ImageJ. With Renewal MI, less than 1% of the image areas were highly fluorescent at either 1 or 14 days. This was significantly lower in comparison to all of the other commercial groups (*p* = 0.01 to 8.3 × 10^−8^). Activa and Z250 gave intermediate results. These were significantly lower than Fuji IX and Fuji II LC, and significantly higher than Renewal MI at day 14.

#### 3.3.3. Mineral Precipitation at Demineralized Dentine/Set Restoration Interfaces

**Figure 8:**
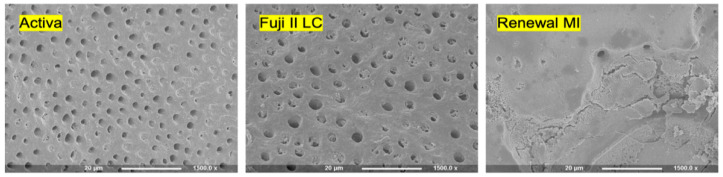
Example SEM images of the collagen mesh discs following their contact with pre-cured Activa, Fuji II LC and Renewal MI discs (*n* = 3 per material). Samples were incubated at 37 °C in SBF for 6 months. Activa and Fuji II LC shows widely opened dentinal tubules. Conversely, Renewal MI caused formation of a layer of minerals that covered the whole surface (*n* = 3).

Collagen discs, incubated with discs of Activa or Fuji II LC for 6 months in SBF, showed no sign of minerals at the interface. Instead, widely opened dentinal tubules were observed. Collagen discs incubated with Renewal MI, however, showed an interface totally covered with minerals, and the dentinal tubules were fully occluded (Figure 8).

## 4. Discussion

This study employed a standard technique for assessing the sealing of sound drilled cavities. It also introduced new methods of investigating fully demineralized dentine stabilization, through penetration and sealing, enzyme deactivation and remineralization. Renewal MI properties were compared with different restorative material classes that have been in clinical use for many years and the newer “bioactive” material, Activa.

### 4.1. Composition of Renewal MI

The experimental composite Renewal MI is urethane dimethacrylate (UDMA)- rather than BISGMA-based, which removes the phthalate (a potential BISGMA impurity) leaching risk [26]. The high molecular weight of its diluent monomer, polypropylene glycol dimethacrylate (PPGDMA), helps to keep shrinkage low. The Renewal MI liquid phase also contains 3 wt% of the adhesion promoting monomer, 4-methacryoyloxy trimellitate anhydride (4META) [24]. Its total filler content is 75 wt% (~55 vol%). It is a hybrid composite containing a mixture of 7 µm- and 0.7 µm-average diameter, radiopaque, silane-treated, barium aluminosilicate glass particles and a lower level of silica nanoparticles.

The food preservative polylysine (PLS) is also included in Renewal MI filler phase at 4 wt% [24]. This component is crucial for enabling composite penetration into dentine tubules [24]. The PLS particles are too large (20–50 µm) to penetrate the tubules themselves, but may act through absorbing water. This could allow the hydrophobic resin phase to penetrate and interact with tooth structure, but additionally lowers strength.

The filler phase of Renewal MI also contains 8 wt% monocalcium phosphate (MCP). In water, MCP can disproportionately form dicalcium phosphate (DCP) and phosphoric acid. The phosphoric acid may, in the presence of surface water, provide self-etch of apatite in tooth structure. The DCP rapidly precipitates as brushite. This process requires water and produces crystals of lower density that occupy greater volume [27]. This can provide expansion of Renewal MI to compensate for polymerization shrinkage or fill gaps at the tooth restoration interface [24,28].

Renewal MI has a high conversion rate and sufficient level and depth of cure (72% at 3 mm depth) for bulk filling without layering [24]. Flow characteristics and polymerization shrinkage (3%) are between those of packable (e.g., Z250) and flowable composites [24,29,30]. The biaxial flexural strength is 120 MPa at 1 day, but declines to 75 MPa at 3–6 months due to water sorption [24]. For comparison, that of Z250 is greater (>150 MPa), those of Activa and Fuji II LC are comparable (100–70 MPa) and that of Fuji IX is weaker (<40 MPa).

### 4.2. Enamel Etching

Enamel prisms can demonstrate one of three etching patterns: demineralized edges, centres of the prisms or a mix of both. Clinically, the pattern has been found to be unimportant, provided there is sufficient roughening to increase the surface area and enable an effective seal [31,32,33].

This study showed that Renewal MI created different etching patterns that also required longer application time to form. MCP in Renewal MI can form phosphoric acid in contact with water as explained above. This acid, however, might provide localized, inhomogeneous hydroxyapatite dissolution primarily near the MCP particles thereby leading to the different enamel roughening patterns. The concentrations of acid produced will be dependent upon levels of moisture and MCP particles at the tooth/composite interface and, as seen in this study, application time. Further work is required to assess whether increasing the time between placement and light exposure can enable improvements in both etch and then bond strengths.

### 4.3. Dye Microleakage in Sound Cavities

Dye microleakage is one of the methods of testing adhesion to permanent teeth detailed in ISO/TS 11405:2015 [22]. In this study, however, primary teeth were employed, since Renewal MI is proposed for paediatric patients. Samples were aged in water at 37 °C for just 24 h to determine early sealing properties. Further work with longer aging and/or thermocycling will be required to assess the effects of long-term interface degradation or mineral precipitation.

In this study, Renewal MI was cured soon after placement, providing limited time for the self-etching properties to enhance interlocking and bond strengths. Furthermore, Renewal MI’s moderate shrinkage did not result in higher microleakage. This contradicts what was expected based on previous studies with other materials [34]. A new early sealing mechanism is possible, maybe due to Renewal MI’s flowability. Additionally, the surface water sorption by PLS and the subsequent absorbed water reaction with MCP and 4META could be important. Tooth surface water removal may enable improved contact and interlocking of the hydrophobic resin phase with the tooth surfaces. Furthermore, the water sorption may promote localized composite swelling to compensate for polymerization shrinkage as the material sets. This sealing could be further improved by water sorption during the 24 h of restoration storage prior to immersion in dye [24]. Further studies will be required to assess if this seal can be maintained in the long term through additional swelling and mineral precipitation to compensate for any loading damage.

The null hypothesis that Renewal MI microleakage is not significantly different from commercial materials can be rejected with the GIC and RMGICs. It cannot, however, be rejected when comparing Renewal MI to Z250 with adhesive. For Z250 (with etch and Scotchbond), microleakage was comparable to Renewal MI and similar to previous observations [35,36]. The high variability in the results with Activa Kids and Fuji II LC suggests the materials may bond to one surface but not the other. The more consistently high leakage with Fuji IX may be due to a more constant gap. The manufacturer, GC, recommends tooth preconditioning with a polyacrylic acid solution and rinsing for both Fuji IX and Fuji II LC. This step, however, was excluded in this study as it is not always employed. Despite this deviation, the dye leakage observed with glass ionomers in this study was similar to previous work in which a conditioner and thermocycling were employed [37]. This suggests that gaps are formed from the start. The variable results with Activa are consistent with clinical study variability. In 1-year clinical studies, Activa (with etch only) gave good results in one study [38], but unacceptably high failure in a different study [39].

### 4.4. Interactions with Demineralized Dentine

In carious dentine, the top surface is infected, degraded, soft, and readily removed. Beneath this, there is an affected, leathery layer which is partially demineralized but repairable [40]. This may cover harder, translucent dentine with tubules occluded by excess minerals. These minerals provide a natural mechanism to protect the underlying pulp. In this study, fully demineralized collagen discs were used to mimic the structure of the intermediate affected dentine layer. Using originally sound teeth, controlling disc thickness and the time of immersion in formic acid enabled the complete removal of the minerals. This provided reproducible standardized model discs that consisted of non-collapsed collagen [24]. These had sound dentinal tubules that could be used to assess collagen tubule sealing by tags or occlusion through precipitation of minerals.

The ability of a restorative material to penetrate and seal caries-affected dentine might improve the interlocking with and strengthening of the diseased tissue. Furthermore, it should reduce water that may promote enzyme catalysed hydrolysis of collagen and consequently limit microleakage and recurrent disease. Additionally, the prevention of nutrient ingress should also cause remnant caries to arrest and provide a favourable environment for remineralization. These features could enable the use of the material without the need for total caries removal or complicated etch and bond procedures.

#### 4.4.1. Resin Tags within Demineralized Dentine

The null hypothesis that Renewal MI tags coverage is not significantly different from commercial materials can be rejected with the GIC and RMGICs. It cannot, however, be rejected when comparing Renewal MI to Z250 with adhesive, despite the considerable difference in tags lengths. While resin tag formation is not by itself a true indicator of the ability of the material to bond to tooth structure, it is an important property to stabilize caries. Renewal MI could penetrate demineralized dentine without any surface pre-treatment and form a dense network of long resin tags. The hydrophilic PLS and MCP particles, together with 4META, have been shown to be primarily responsible for the high-dentine surface coverage [24]. These components may absorb water from the dentinal tubules, creating space for the fluid paste to penetrate. The patchy tag-free areas may be due to remnant water blocking tubules. The length of the tags is mainly controlled by the fluidity of the paste rather than hydrophilic component levels. Lower filler content enables faster and thereby greater movement into the tubules [24]. The resin tags formed by Renewal MI within demineralized dentine were much longer (>200 µm) than those reported in the literature. Typically, adhesive resin tags in acid-treated dentine are 20–30 µm [41,42]. Further work is needed, however, to analyse the chemical composition of these resin tags.

Preliminary studies showed that the conventional composite Z250 formed no tags on its own due to its high viscosity and hydrophobicity [24]. The observation of tags when Z250 was used with Scotchbond could be a consequence of the hydrophilic adhesive. The high fluidity of Scotchbond should encourage fast flow into the tubules. The small amount of adhesive applied combined with its BISGMA content, however, might limit the amount of water that could be absorbed. This might explain the shorter, less dense resin tags of Scotchbond compared with Renewal MI.

The flowability of Activa in combination with hydrophilic components might explain its ability to form resin tags with demineralized dentine. The lower surface coverage than that seen with Renewal MI, however, could be a consequence of Activa’s lesser water sorption ability.

Whilst Fuji II LC and Fuji IX can both absorb water, their high viscosities might restrict their ability to penetrate the demineralized dentine. Furthermore, the large size of the polyacids in their liquid phase and interactions with positively charged amino acids in the dentine might further slow the rate of flow. Instead, a distinct few micron-thick water insoluble layer is known to form at the tooth interface [43].

#### 4.4.2. Enzyme Activity in Restored Demineralized Dentine

The molecular probe used in this study provided a layer of high fluorescence on the surface of the control sample. This fluorescence will have been due to hydrolysis of collagen particles catalysed by enzymes activated in the demineralized dentine surface. The limited depth of fluorescence could be a consequence of the probe failing to diffuse into the dentine bulk. Alternatively, enzymes may only be activated near the dentine surface. Reduction in the fluorescence after 2 weeks might have arisen through the surface layer collapsing following enzyme-catalysed collagen hydrolysis.

The null hypothesis that Renewal MI enzyme inhibition is not significantly different from all commercial comparators can be rejected at day 1 and 14. For restored discs, the fluorescence area at the adhesion interface correlated with level of tags formation. The reduction in fluorescence area at 2 weeks with Renewal MI might be a consequence of it absorbing the remaining water and improving the seal by forming resin tags.

Similarly, the Scotchbond adhesive used with Z250 might have provided an effective seal. With the other restorative materials, larger interfacial gaps containing water and the molecular probe might have caused both the higher area of interface fluorescence and lesser area reduction with time.

The importance of good dentine sealing on inhibiting MMP activity has previously been addressed in the literature [44,45]. Low pH caused by the natural caries process (bacterial acids) or acid-etching can result in activation of the MMP enzymes. This could be responsible for future degradation and nanoleakage at the interface, leading to recurrence of the disease and failure of the restoration. Research has shown that host-derived MMPs combined with cysteine cathepsins are the most abundant and active enzymes in carious dentine. Furthermore, MMP release from collagen is a key feature for caries progression [40,46,47,48,49].

### 4.5. Mineral Precipitation in Demineralized Dentine

The above observed ability of Renewal MI to precipitate minerals, at the demineralized dentine interface, can be explained by the MCP content. The hydrophilic MCP can interact with water as explained above to form phosphoric acid and dicalcium phosphate. The release of these from the composite will provide calcium and phosphate ions. These may supersaturate the storage solution (SBF), resulting in the precipitation of various calcium phosphate minerals. The amount of remineralization thickness and occluding dentinal tubules looks more promising in comparison to a casein phosphopeptide-amorphous calcium phosphate (CPP-ACP)-containing paste [50]. The lack of mineral precipitation with Activa and Fuji II LC suggests that their release of ions is insufficient to supersaturate the SBF. Further analysis of the chemical composition of the precipitated layer at the interface between Renewal MI and dentine is needed.

## 5. Conclusions

Renewal MI can self-etch enamel. Its early ability to seal drilled cavities is comparable with Z250 (with Scotchbond) and superior to that of Activa, Fuji II LC and Fuji IX. With demineralized dentine, Renewal MI gave denser and longer resin tags and reduced enzyme activity. Unlike Activa or Fuji II LC, it promoted the precipitation of minerals.

## 6. Patents

A.Y has two patents on the use of MCP and PLS in dental composites licensed to a dental company (Davis Schottlander and Davis Ltd., Letchworth Garden City, UK).

## Figures and Tables

**Table 1:** Main components in materials under investigation according to manufacturers.

Material	Type	Manufacturer	Composition	
			Liquid Phase	Filler
RENEWAL MI	Single step experimental flowable hybrid composite	Davis, Schottlander and Davis Dental Company, Letchworth, UK	UDMA, PPGDMA, 4META, CQ	Strontium alumino silicate, Monocalcium phosphate, Polylysine
Filtek Z250 (Shade B3)	Conventional composite	3M ESPE, St. Paul, MN, USA	BISGMA, UDMA, other dimethacrylates	Zirconia/silica (0.6 µm)
Scotchbond Universal	Combined selective etch, self-etch, total etch	3M ESPE, St. Paul, MN, USA	BISGMA, HEMA, 10MDP, methacrylate modified polyacid, water, ethanol	Silica
Activa kids (Pedo shade double-barrel syringe)	Bioactive RMGIC	Pulpdent, Watertown, MA, USA	UDMA, other methacrylates, modified polyacid	Reactive and unreactive fillers, sodium fluoride
Fuji II LC (Shade A2 capsules)	RMGIC	GC America, Alsip, IL, USA	HEMA, UDMA, polyacid, water	Silane treated strontium fluoroaluminosilicate
Fuji IX GP (Shade A2 capsules)	GIC	GC America, Alsip, IL, USA	Polyacid, water	Strontium fluoroaluminosilicate

Abbreviations: RMGIC (resin-modified glass ionomer cement), GIC (glass ionomer cement), UDMA (urethane dimethacrylate), PPGDMA (polypropylene glycol dimethacrylate), 4META (4-methacryoyloxy trimellitate anhydride), CQ (camphorquinone), BISGMA (bisphenol a-glycidyl methacrylate), HEMA (Hydroxyethylmethacrylate), 10MDP (10-methacryloyloxydecyl dihydrogen phosphate).

## Data Availability

The data presented in this study are available on request from the corresponding author.
